# Association of Polygenic Liability for Alcohol Dependence and EEG Connectivity in Adolescence and Young Adulthood

**DOI:** 10.3390/brainsci9100280

**Published:** 2019-10-17

**Authors:** Jacquelyn L. Meyers, David B. Chorlian, Emma C. Johnson, Ashwini K. Pandey, Chella Kamarajan, Jessica E. Salvatore, Fazil Aliev, Stacey Subbie-Saenz de Viteri, Jian Zhang, Michael Chao, Manav Kapoor, Victor Hesselbrock, John Kramer, Samuel Kuperman, John Nurnberger, Jay Tischfield, Alison Goate, Tatiana Foroud, Danielle M. Dick, Howard J. Edenberg, Arpana Agrawal, Bernice Porjesz

**Affiliations:** 1 Department of Psychiatry, State University of New York Downstate Medical Center, Brooklyn, NY 11203, USA; 2 Department of Psychiatry, Washington University School of Medicine, St. Louis, MO 63110, USA; 3 Department of Psychology, Virginia Commonwealth University, Richmond, VA 23284, USA; 4 Virginia Institute for Psychiatric and Behavioral Genetics, Virginia Commonwealth University, Richmond, VA 23284, USA; 5 Department of Neuroscience, Icahn School of Medicine at Mount Sinai, New York, NY 10029, USA; 6 Department of Psychiatry, University of Connecticut School of Medicine, Farmington, CT 06030, USA; 7 Department of Psychiatry, Roy J and Lucille A Carver College of Medicine, University of Iowa, Iowa City, IA 52242, USA; 8 Department of Psychiatry, Indiana University School of Medicine, Indianapolis, IN 46202, USA; 9 Department of Genetics and the Human Genetics Institute of New Jersey, Rutgers University, Newark, NJ 08901, USA; 10 Department of Genetics and Genomic Sciences, Icahn School of Medicine at Mount Sinai, New York, NY 10029, USA; 11 Department of Medical and Molecular Genetics, Indiana University School of Medicine, Indianapolis, IN 46202, USA; 12 Department of Biochemistry and Molecular Biology, Indiana University School of Medicine, Indianapolis, IN, 46202, USA

**Keywords:** AUD, AD, PRS, neural connectivity, EEG coherence, sex differences, developmental trajectories

## Abstract

Differences in the connectivity of large-scale functional brain networks among individuals with alcohol use disorders (AUD), as well as those at risk for AUD, point to dysfunctional neural communication and related cognitive impairments. In this study, we examined how polygenic risk scores (PRS), derived from a recent GWAS of DSM-IV Alcohol Dependence (AD) conducted by the Psychiatric Genomics Consortium, relate to longitudinal measures of interhemispheric and intrahemispheric EEG connectivity (alpha, theta, and beta frequencies) in adolescent and young adult offspring from the Collaborative Study on the Genetics of Alcoholism (COGA) assessed between ages 12 and 31. Our findings indicate that AD PRS (*p*-threshold < 0.001) was associated with increased fronto-central, tempo-parietal, centro-parietal, and parietal-occipital interhemispheric theta and alpha connectivity in males only from ages 18–31 (beta coefficients ranged from 0.02–0.06, *p*-values ranged from 10^−6^–10^−12^), but not in females. Individuals with higher AD PRS also demonstrated more performance deficits on neuropsychological tasks (Tower of London task, visual span test) as well as increased risk for lifetime DSM-5 alcohol and opioid use disorders. We conclude that measures of neural connectivity, together with neurocognitive performance and substance use behavior, can be used to further understanding of how genetic risk variants from large GWAS of AUD may influence brain function. In addition, these data indicate the importance of examining sex and developmental effects, which otherwise may be masked. Understanding of neural mechanisms linking genetic variants emerging from GWAS to risk for AUD throughout development may help to identify specific points when neurocognitive prevention and intervention efforts may be most effective.

## 1. Introduction

Alcohol use disorder (AUD) results from a complex interaction of genetic and environmental liabilities across the lifespan [1]. Variations in brain structure and function are both antecedents to AUD and consequences of the effects of excessive alcohol consumption [2]. Differences in the connectivity of large-scale brain networks are observed among individuals with neuropsychiatric disorders, including AUD, and in their offspring [3]. Given its exquisite temporal resolution (millisecond range), EEG coherence has proven to be a useful measure of neural connectivity patterns in specific EEG frequency bands which, alongside clinical and fMRI data with spatial resolution allowing for anatomical specificity, can further our understanding of neural mechanisms of AUD [4]. Research characterizing genetic variation for AUD with respect to measures of neural connectivity can advance understanding of how genomic risk for AUD affects neurocognitive function.

Recently, the number of large genome-wide association studies (GWAS) of alcohol-related behaviors has increased, covering a spectrum of phenotypes ranging from alcohol use to AUD diagnoses. It has become increasingly clear that although alcohol consumption and AUD are genetically related, they do not have the same genetic architecture [5,6,7,8]. Importantly, AUD shares genetic liability with other psychiatric comorbidities, whereas alcohol consumption does not [5,6]. Several parallel efforts are underway to identify genes associated with alcohol problems and clinical AUD diagnoses. A GWAS of DSM-IV alcohol dependence (*N* = 52,848) conducted by the Psychiatric Genomics Consortium identified variants in *ADH1B* [8]. A 2019 meta-analysis of two population-based studies of the Alcohol Use Disorder Identification Test (AUDIT; *N* = 141,932 individuals) [5] replicated previously identified signals in the genes *ADH1B, ADH1C, KLB*, and *GCKR* and identified novel associations localized to genes, including *JCAD* and *SLC39A13*. More recently, 18 genome-wide significant loci were identified in a GWAS of alcohol use disorder from the Million Veteran Program [6]. Forthcoming findings from even larger consortia-based efforts will be highly informative for gene discovery regarding alcohol use behavior and AUD. However, GWAS represents only the beginning of our understanding of genomic influences on AUD. Deeply phenotyped samples are needed to characterize results emerging from their findings, particularly with respect to brain function, which might serve as a pathway for genetic vulnerability for AUD to be expressed. Polygenic risk scores (PRS) aggregate genetic information from GWAS [9], permitting studies of aggregated risk in independent samples. Briefly, PRS represent an individual’s genetic liability for a trait or disorder such as alcohol dependence, created by summing the effect sizes (from a GWAS) of many risk variants for the phenotype of interest, weighted by the number of effect alleles in individual carriers at each locus. By harnessing the well-known and massive polygenicity underlying AUD, PRS can be used to enhance our understanding of how genetic vulnerability to AUD is reflected in neural connectivity, among other aspects of brain activity, and thus provide important context to findings emerging from these large-scale genomic studies.

EEG coherence is the degree of synchrony in brain oscillatory activity between neural networks in two brain regions [10,11,12]. Stated another way, increased coherence between two EEG electrodes suggests functional integration of the two brain regions, whereas decreased coherence reflects the unrelated activities of two neural populations [13,14]. Inter-hemispheric coherence measures synchrony of contralateral neural activity (between the right and left hemispheres in the same brain regions), mainly attributed to connectivity between the two hemispheres via the corpus callosum, whereas intra-hemispheric coherence measures the degree of coupling across brain regions, including longer range cortico-cortical connectivity patterns (e.g., between frontal and parietal brain regions). EEG coherence utilizes temporal resolution on the order of milliseconds, the scale at which most relevant sensory, motor, and cognitive phenomena take place at the neural level [15]. It is therefore able to assess local and distal connectivity patterns as a function of frequency band, as aspects of neural function and connectivity patterns between brain regions are specific to EEG frequency [15]. While research illuminating precise neurobehavioral correlates of EEG coherence has been limited, differences in EEG coherence have been observed with a variety of cognitive processes such as emotion recognition [16], social interactions [17], working memory [18], various aspects of intelligence [19,20], and aspects of cognitive impairment [21,22]. It also has been used to index brain maturation [13,22,23]. Importantly, Chorlian and colleagues [24] reported frequency-specific topographical patterns in bipolar EEG coherence and found an interesting similarity of these patterns with those obtained by resting state networks identified by fMRI studies.

While EEG coherence has been examined in various neuropsychiatric conditions—including attention deficit hyperactivity disorder [25], Alzheimer’s dementia [26], autism [27], and schizophrenia [28]—there have been a limited number of large, well-powered studies that have examined EEG coherences in individuals with and without AUD. In the studies that have been conducted, there seems to be consensus that for theta (4–7 Hz), alpha (8–12 Hz), and beta (12–28 Hz) frequencies, local resting state coherences are increased among adults with AUD. Winterer and colleagues [29] reported that intra-hemispheric posterior coherences were increased in the alpha and beta bands in both long-term abstinent and non-abstinent alcohol-dependent study participants. Similarly, Porjesz and Rangaswamy [30] report higher interhemispheric coherence in most frequency bands (theta, alpha, beta) in an age-matched sample of abstinent alcohol dependent participants and controls, particularly posteriorly at parietal-occipital and centroparietal regions in theta band. More recently, Cardenas et al. [4] have reported increased alpha and theta coherence, particularly at central, parietal, and occipital electrodes in long term abstinent alcohol dependent individuals. In the Collaborative Study on the Genetics of Alcoholism (COGA) parietal-occipital theta coherence was found to be associated with key alcohol candidate genes, *GABRA2* and *CHRM2* [30]. EEG coherence is known to change dynamically across the lifespan, with some studies of the general population showing increases in childhood [31], adolescence and early adulthood [32] and decreases in later adulthood [33]. Thus, coherence has been particularly useful in the study of normal brain development and neuropsychiatric disorders such as AUD [34]. Furthermore, gender differences have been observed for resting EEG coherence in both adults and children, typically with females displaying higher coherence levels across frequencies [35,36], underscoring the complexity of neural development and the importance of examining these effects in both males and females.

Adolescence and young adulthood is an extremely important developmental period, given the critical brain maturation occurring [37,38] alongside typical experimentation with alcohol and other drugs [39]. Genomically informed longitudinal, developmental AUD research incorporating measures of brain function would provide an important neurodevelopmental perspective. Given the increased risk for AUD among those with a family history of AUD, studying developmental trajectories of brain function in adolescents and young adults from families affected with AUD would enable the identification of molecular genetic factors that increase the likelihood of developing an AUD. Although there have been longitudinal studies examining alcohol use behavior and brain function in adolescents and young adults—notably in the ALSPAC study [40], ABCD study [41], and NCANDA [42]—genomically informed longitudinal AUD research incorporating measures of brain function is lacking, particularly in adolescents and young adults at high risk for AUD.

As part of the Collaborative Study on the Genetics of Alcoholism (COGA), longitudinal EEG coherence data were collected on adolescent and young adult offspring ages 12–31 from families densely affected with AUD as well as community comparison families. In the current study, we examined whether polygenic risk for DSM-IV Alcohol Dependence, based on summary statistics from the meta-analysis conducted by the PGC [8], is reflected in individual differences in theta, alpha, and beta EEG coherence among adolescents and young adults throughout a key period of risk for the onset of AUD (ages 12–31). Furthermore, we examined how these associations differ between males and females to identify sex- and age-specific effects that may be otherwise masked in cross-sectional samples that combine across age and sex.

## 2. Methods

### 2.1. Sample and Measures

COGA’s prospective study began data collection in 2004 and ended in 2019. Details on data collection and procedures have been published previously [43]. Briefly, offspring from families densely affected with AUD and comparison community families who were aged 12–22 at intake, and who had at least one parent interviewed in an earlier phase of the COGA study, were enrolled, with new subjects added as they reached the age of 12. Subjects were assessed every two years with a comprehensive battery that includes the Semi-Structured Assessment for the Genetics of Alcoholism (SSAGA) [44,45] assessing substance use problems as well as other psychiatric disorders and related behaviors, personality questionnaires, neurocognitive performance, and a neurophysiological battery that includes resting state EEG. The sample comprises 2625 offspring from 2413 nuclear families. Of the 2625 offspring (1286 males and 1339 females), 1931 had at least one follow-up interview, 1324 had two follow-up interviews, 842 had three follow-up interviews, 428 had four follow-up interviews, and 8 had five follow-up interviews at the time of analysis. The analytic sample in the present study comprises all offspring who had complete EEG coherence data. Due to the majority European ancestral composition of the PGC AD GWAS, once COGA participants were removed, only individuals of European ancestry were included in this study (*N* = 1426 offspring from 913 European American nuclear families who had at least one follow-up interview, total number of observations = 5006). The number of observations available in the current study by age and sex are provided in Supplemental Figure S1. Among this analytic sample, the mean age at first interview was 17.1 (SD = 3.6, Range = 12–26), 51.6% were female, and self-reported ‘race’ was 98.1% European American, 0.1% African American, 0.1% Latin/Hispanic, and 4.4% ‘other’. 41% had a family history of AUD. Experimental protocols were approved by each site’s institutional review board, and informed consent was obtained from all participants. Parental permission was obtained for all participants under the age of 18.

Genotyping for the COGA European American participants was performed using the Illumina 1M, Illumina OmniExpress, and Illumina 2.5 M (Illumina, San Diego, CA, USA), and Smokescreen (BioRelm, Walnut, CA, USA) arrays. Array type was included as a covariate in all analyses. A pruned set of 47,000 variants that were genotyped on all platforms and had minor allele frequencies > 10% in the combined samples, Hardy–Weinberg equilibrium (HWE) *p*-values > 0.001, missing rates < 2%, and were not in linkage disequilibrium (LD, defined as *R*^2^ < 0.5) were used to assess reported pedigree structure using identity-by-descent calculations in PLINK [46]. Family structures were altered as needed and SNP genotypes were tested for Mendelian inconsistencies with the revised family structure [47]. Genotype inconsistencies were set to missing. Imputation was to 1000 Genomes (EUR and AFR, Phase 3, b37, October 2014; build hg19) using SHAPEIT2 [48] and then Minimac3 [49]. Imputed SNPs with information (INFO) scores < 0.30 or individual genotype probability scores < 0.90 were excluded, as were palindromic SNPs (A/T or C/G), monomorphic SNPs, SNPs with a genotyping rate of < 95%, SNPs that were not in HWE (*p* < 1 × 10^−6^), and SNPs with a minor allele frequency less than 0.05%. In total, 6,881,872 SNPs passed quality control and were available for analysis.

Polygenic risk scores (PRS) were derived from the Walters et al. [8] GWAS meta-analysis, after removal of COGA subjects, leaving 9455 alcohol dependent cases and 27,979 controls. We selected the AD GWAS because it was based on a well-defined and clinically-relevant lifetime measure of alcohol dependence, in contrast to the GWAS of the Alcohol Use Disorders Identification Test [50] which relied on scores for problem use based on recent (previous year) patterns of alcohol intake. Given the current study’s goal to investigate sex differences in the influence of AD PRS on neural connectivity development, the largely male Million Veterans Program GWAS, may not have been appropriate. Regardless, at the time of these analyses, summary statistics from the Million Veterans Program GWAS of AUD were not available for use. PRS were coded for every individual in COGA by multiplying an individual’s number of risk alleles (see thresholds below) by the negative logarithm (base 10) of the *p*-value and by the sign of the effect size. Clumping was done with respect to the linkage disequilibrium (LD) pattern in the COGA European ancestry sample (founders only) using a 500 kb physical distance and an LD threshold of *r*^2^ ≥ 0.10. Scores representing effect sizes with nine decreasing thresholds of statistical significance in the discovery GWAS were constructed (*P* < 0.0001, *P* < 0.001, *P* < 0.01, *P* < 0.05, *P* < 0.1, *P* < 0.2, *P* < 0.3, *P* < 0.4, *P* < 0.5). For subsequent use in association analysis, PRS values were *z*-scored. To aid in the interpretation, PRS values were *z*-scored so that effect sizes could be expressed as standard deviations of coherence values per standard deviation of PRS.

**Figure 1:**
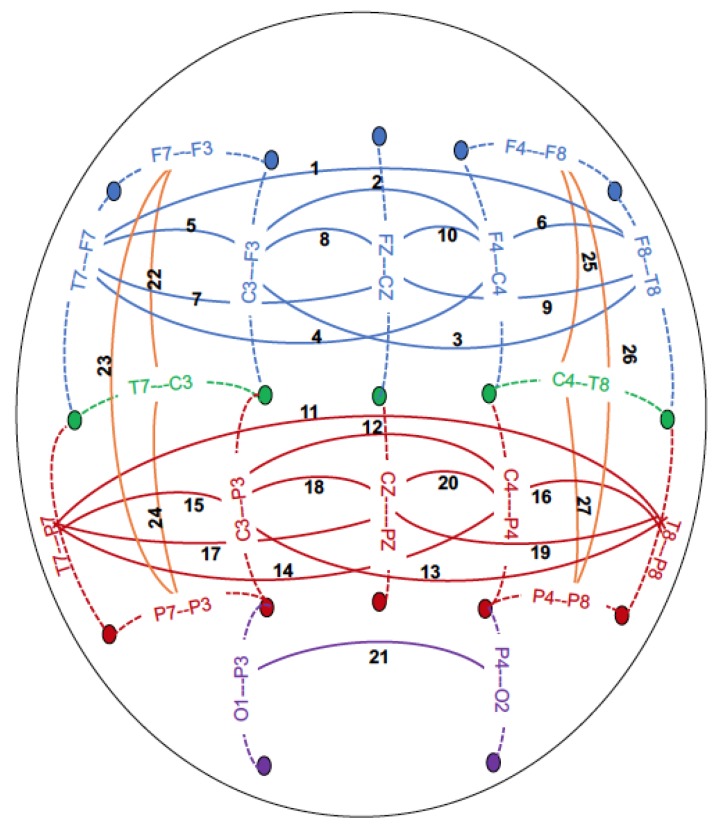
Schematic of the bipolar electrode pairs (connected with dotted lines) and coherence pairs (connected with solid lines) derived between bipolar electrode pairs. Frontal-central sagittal coherence pairs are represented in blue; (**1**) F8-T8–F7-T7, (**2**) F4-C4–F3-C3, (**3**) F3-C3–F8-T8, (**4**) F4-C4–F7-T7, (**5**) F3-C3–F7-T7, (**6**) F4-C4–F8-T8, (**7**) FZ-CZ–F7-T7, (**8**) FZ-CZ–F3-C3, (**9**) FZ-CZ–F8-T8, (**10**) FZ-CZ–F4-C4. Central-parietal sagittal coherence pairs are represented in red; (**11**) T8-P8–T7-P7, (**12**) C4-P4–C3-P3, (**13**) C3-P3–T8-P8, (**14**) C4-P4–T7-P7, (**15**) C3-P3–T7-P7, (**16**) C4-P4–T8-P8, (**17**) T7-P7–CZ-PZ, (**18**) C3-P3–CZ-PZ, (**19**) T8-P8–CZ-PZ, (**20**) C4-P4–CZ-PZ. Parietal-occipital sagittal coherence pairs are represented in purple; (**21**) P4-O2–P3-O1. Intra-hemispheric lateral coherence pairs are represented in orange; (**22**) T7-C3–F7-F3, (**23**) P7-P3–F7-F3, (**24**) P7-P3–T7-C3, (**25**) T8-C4–F8-F4, (**26**) P8-P4–F8-F4, (**27**) P8-P4–T8-C4.

EEG recording and processing have been detailed previously [24]. Briefly, resting (eyes-closed) EEG was recorded for 4.25 min; a continuous interval of 256 s was analyzed. Each subject wore a fitted electrode cap (Electro-Cap International Inc.; Eaton, OH) using the 19-channel montage as specified according to the 10–20 International system (FP1, FP2, F7, F3, FZ, F4, F8, T7, C3, CZ, C4, T8, P7, P3, PZ, P4, P8, O1, O2). The nose served as reference and the ground electrode was placed on the forehead. Electrode impedances were always maintained below 5 KOhm. EEG was recorded with the subjects seated comfortably in a dimly lit sound-attenuated temperature-regulated booth (Industrial Acoustics Company; Bronx, NY, USA). They were instructed to keep their eyes closed and remain relaxed, but not to fall asleep. Electrical activity was amplified 10,000 times by Neuroscan amplifiers, with a bandpass between 0.02 Hz to 100 Hz and recorded using the Neuroscan software system (Compumedics Limited; El Paso, TX, USA) running on i86 PCs. EEG procedures were identical at all collection sites. Further details regarding the EEG coherence data processing is provided in the Supplemental Material. Bipolar electrode pairs were derived to reduce volume conduction effects [11], and the 27 coherence pairs used in this study (depicted in Figure 1) were selected based on a previous study of EEG coherence [24]. Conventional Fourier transform methods [11] were used to calculate coherence. Coherence measures were generated at the following frequencies: low theta (3–5 Hz), high theta (5.5–7 Hz), low alpha (7.5–9 Hz), high alpha (9.5–12 Hz), low beta (12.5–16 Hz), mid beta (16.5–20 Hz), and high beta (20.5–28 Hz) and between pairs of bipolar derivations as described in Figure 1.

Computerized versions of the Tower of London test (TOLT) and the visual span test (VST) were administered using the Colorado Assessment Tests for cognitive and neuropsychological assessment [51]. The TOLT assesses planning and problem-solving ability [52], and the VST assesses visuospatial memory span and working memory. Both tasks have been detailed in previous work [53]. Briefly, in the TOLT, participants were required to plan a sequence of moves to go from the starting position of a set of colored beads to a goal position in as few moves as possible and execute the moves one-by-one with the following constraints: only one bead at a time may be moved off a peg; and beads may not be left off pegs. The test comprised of three 2-move, and six each of 3-, 4-, and 5-move problems for a total of 21 problems. TOLT performance (number of trials completed with optimal performance) and speed (average trial time) were analyzed. In the VST, participants were presented with eight boxes illuminated on a screen in specific sequences and were asked to designate the sequence (which might be forward or backward). The task began with a sequence of two boxes, and this was maintained until the participant missed two trials or correctly performed up to eight sequences. VST performance on backwards and forward sequences were analyzed.

### 2.2. Statistical Analysis

We examined the association of AD PRS (nine *p*-value thresholds) with developmental trajectories of interhemispheric and intrahemispheric EEG coherence (low theta, high theta, low alpha, high alpha, low beta, mid beta, high beta) in the sample described above. Correlations between AD PRS (Supplemental Table S1) indicated that only the three most stringent AD PRS were independent (the seven least stringent PRS (*P* < 0.01, *P* < 0.05, *P* < 0.1, *P* < 0.2, *P* < 0.3, *P* < 0.4, *P* < 0.5) were redundant); therefore, only PRS *p* < 0.0001, *p* < 0.001, *p* < 0.01 examined in this study.

Association of PRS with EEG coherence trajectories were calculated as described in detail in Chorlian et al. [54]. Briefly, to test for association between each PRS and EEG coherence phenotype (27 coherence pairs, 7 frequency bands, 3 PRS levels), a local linear (non-parametric) regression model was calculated, including the PRS as the predictor and the inverse hyperbolic tangent of the coherence measure as the dependent variable. Using an additive model, 96 age centers, 2/10ths of a year apart from ages 12 to 31, were utilized to estimate the age specific association of the PRS with EEG coherence phenotypes, using an additive model. An Epanechnikov kernel with a bandwidth of 0.6 was utilized. Since this method uses sliding age windows, there was considerable overlap in the data used in calculations for nearby ages.

In view of the critical need to systematically examine sex differences in genetically informed studies of AUD [55], sex differences previously observed in EEG measures in the analytic sample [56], and to correspond to methods presented in other genetic studies of development [57], sex specific models were utilized. Covariates in the model included family history of AUD, ancestral principal components (PC1-PC3), and a sex × PRS interaction term. Weights for individuals were adjusted to account for multiple observations on single individuals and co-presence of related individuals (e.g., siblings) in each of the local linear regression calculations.

Next, we selected significant PRS and frequency band pairs (low theta, high theta, low alpha, high alpha, low beta, mid beta, high beta) for subsequent investigation. This selection was performed separately among males and females. For each frequency band, and PRS, results from the 96 associations of the PRS and EEG coherence pairs were divided into three age groups (12–18, 18–25, 25–31). For each age group, the first-quartile *p*-value was calculated. PRS and frequency bands were selected when at least eight EEG coherence pairs with *p*-values < 10^−6^ were present in at least one of the three age ranges. The use of the first quartile *p*-value is a conservative method to avoid isolated false positives and to ensure biological meaningfulness in a procedure which produces large numbers of correlated results because of the use of overlapping data sets, and the relatively high degree of phenotypic correlation among EEG coherences. Among the frequency bands that met this stringent criteria, the entire association time series of each EEG coherence pair and PRS was examined, and a false discovery rate estimation was applied. All analyses were conducted in MATLAB.

In secondary analyses, we examined the association of the AD PRS (for only the single most significant *p*-value threshold as selected on the basis of the EEG coherence association findings) with neuropsychological performance, including planning and problem-solving skills on the Tower of London task and visuospatial memory and working memory on the visual span test (forwards span and backwards span), and DSM-5 symptom counts (lifetime) for alcohol, cannabis, cocaine, and opioid use disorder. We also conducted sensitivity analyses in which all models were re-examined with the inclusion of alcohol consumption (i.e., maximum number of drinks consumed in a typical week, past 12 months) and DSM-5 AUD diagnosis in the model to determine if the effects of alcohol consumption and/or AUD were driving associations. Finally, we re-ran the model stratified by those with and without a DSM-5 AUD diagnosis (lifetime) to determine if developmental patterns of PRS-EEG coherence associations differed among these groups.

## 3. Results

While 71.8% of the analytic sample had ever consumed alcohol in their lifetime, 35.4% met criteria for DSM-5 AUD, 23.4% met criteria for DSM-5 cannabis use disorder, 4.2% met criteria for DSM-5 cocaine use disorder, and 5.3% met criteria for DSM-5 opioid use disorder (Table 1). The average maximum number of drinks consumed in a typical week (past 12 months) was 6.6 (SD = 12.1).

**Figure 2:**
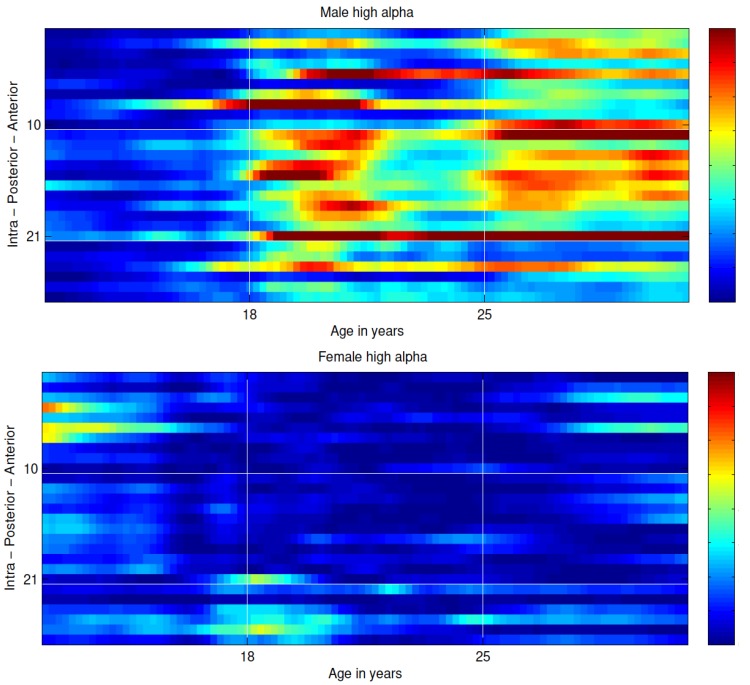
Association (−log10 *p*-value) of DSM-IV AD PRS (*p* < 0.001 threshold) at coherence pairs (*y*-axis) organized (top to bottom) from interhemispheric anterior pairs to posterior pairs, and intra-hemispheric pairs; prominent associations with fronto-central, tempo-parietal, centro-parietal, and parietal-occipital interhemispheric high alpha coherence are observed only among males from ages 18–31.

AD PRS (*p*-threshold < 0.001) was associated with *increased* EEG coherence in COGA participants (low theta, high theta, low alpha, high alpha). Only associations in low theta and high alpha frequency bands with PRS threshold *p* < 0.001 in males met the initial selection criterion, with the majority of coherence pairs in these frequency bands showing strong association with AD PRS in ages 18–31 (effect sizes ranged from 0.15 to 0.21 with all *p*-values < 10^−4^, see Table 2 and Supplemental Figure S2). The most robust associations were observed in fronto-central (FZ-CZ–F3-C3), temporo-parietal (T8-P8–T7-P7), centro-parietal (C4-P4–C3-P3, C3-P3–CZ-PZ), and parieto-occipital (P4-O2–P3-O1) coherences; *p*-values ranged from 5 × 10^−5^ through 5 × 10^−8^ (Figure 2, top panel). A false discovery rate estimation for correlated data with a criterion of 10^−3^ was applied to the entirety of the results for low theta and high alpha (2592 values per band). For low theta, associations with *p*-values< 3.2 × 10^−4^ met this criterion and for high alpha associations with *p*-values < 3.9 × 10^−4^ met this criterion (Table 2). Additionally, robust associations were also observed in high theta and low alpha frequencies with PRS threshold *p* < 0.001 in males (Supplemental Table S2, Supplemental Figure S3) but not in enough coherence pairs to meet our stringent selection criteria. No robust associations of PRS and EEG coherence were observed among females, regardless of age, frequency band, and PRS *p*-value threshold (Figure 2, bottom panel; Supplemental Table S3). Significant PRS × sex interactions were observed for the majority of the significant associations reported in males (Supplemental Table S4).

Sensitivity models were only examined in males. PRS-EEG coherence association models including maximum number of drinks consumed in a typical week and DSM-5 AUD as covariates, results were largely unchanged (Supplemental Tables S5 and S6, respectively). Models stratified by DSM-5 AUD diagnosis show that the patterns of PRS-EEG coherence associations differed among the two groups; among those with alcohol use disorder, prominent associations with fronto-central, tempo-frontal (F3-C3–F7-T7, FZ-CZ–F3-C3), centro-parietal (C4-P4–C3-P3, C3-P3–CZ-PZ), parietal-occipital (P4-O2–P3-O1), and fronto-parietal (P8-P4–F8-F4) high alpha coherences are observed only among males from ages 18–24 (Supplemental Figure S4, panel A), whereas prominent associations are only observed with parietal-occipital (P4-O2–P3-O1) high alpha coherences among males from ages 24–31 (Supplemental Figure S4, panel B).

Individuals with higher AD PRS (*p* < 0.001 threshold) also demonstrated deficits in neuropsychological performance, including poorer planning and problem solving skills on the Tower of London task (*B*: −0.097, *p* < 0.001) and visuospatial memory and working memory on the visual span test (forwards span *B*: −0.123, *p* < 0.001), and had an increased number of symptoms for DSM-5 AUD (*B*: 0.087, *p* < 0.001) and opioid use disorder (*B*: 0.069, *p* < 0.001), but not cannabis or cocaine use disorders (*B*: 0.43, *p* > 0.1, *B*: 0.037, *p* > 0.1 respectively). While the magnitude of these associations was reduced when maximum number of drinks per week were included in regression models, all associations remained significant (Table 3).

## 4. Discussion

In this study, we examined how PRS derived from a recent GWAS of DSM-IV AD [8] is associated with development of interhemispheric and intrahemispheric EEG coherence. AD PRS were associated with frontal-central, temporal-parietal, and parietal-occipital theta and alpha connectivity in adolescent and young adult COGA participants; this association was highly pronounced in males from ages 18–31, but not in females. Individuals with higher AD PRS also demonstrated deficits in neuropsychological performance, and increased risk for alcohol and opioid use disorders. When drinking frequency was controlled for, all associations remained statistically significant, indicating that the influence of the AD PRS on neural connectivity observed in this study is not primarily the effect of alcohol use on the brain. From these data, we conclude that measures of neural connectivity can be used to further understanding of how polygenic liability to AD may influence brain function, as well as the importance of examining sex and developmental effects in polygenic associations. We note however, that effect sizes were modest, ranging between 0.15 and 0.21 (beta coefficients ranged from 0.02–0.06), consistent with other developmental studies of EEG phenotypes [54,58].

While this is the first study to our knowledge that has examined the influence of any neuropsychiatric PRS on EEG coherence, previous research has demonstrated increases in resting state interhemispheric coherence among individuals with AUD, most prominent in theta, alpha band, indicating altered thalamo-cortical and cortico-cortical functional connectivity, and less prominent in beta bands [4,29,30]. As beta coherence values are (generally) considerably lower than theta and alpha values, and coherence is an absolute scale (rather than a ratio scale), the low level of beta coherence values may contribute to the lack of associations observed in this and other studies. In the current study, we found the most prominent effects concentrated in interhemispheric central-parietal pairs, extending to frontal and temporal pairs and across low frequency bands, with increases in coherences corresponding to increases in PRS. Recent work [4] identified resting state theta and alpha EEG coherence networks that were correlated with resting state executive control networks detected with fMRI in prior AD studies; when compared to light drinkers, individuals with AUD have reduced cortical grey matter [59,60] and white matter volumes, and reduced myelination [61,62].

The brain undergoes its greatest growth and development in the first years of life, with a second phase beginning in adolescence characterized by growth of white matter tracts and synaptic pruning in gray matter, leading to anatomical and functional maturation [63,64,65,66]. This second phase of development is most profound in the prefrontal cortex and its connections to limbic structures—regions of the brain involved in higher order cognitive functions (e.g., top-down control functions, such as inhibition and other aspects of executive function), as well as affective functions (e.g., emotional and reward processing; [63,67]). In data from this study, we see important changes in the development of EEG coherence, most notably the increases in coherence from ages 12–18 occurring in fronto-temporal and centro-parietal coherence before a leveling-off observed in the early 20s, and the decreases observed in fronto-central coherence after age 20 in some locations and frequency bands (Supplemental Figure S5). These developmental changes may reflect increasing white matter known to occur in the early 20s [68]. From data observed in other developmental studies [69] measured at shorter time intervals than those in the current study, it is likely that individuals change more rapidly than what can be observed by mean trajectories over brief time intervals, i.e., there are growth spurts in an individual’s neural development. Thus, developmental differences in the influence of AD PRS on EEG coherence observed in this study may reflect genetic influences on coherence growth spurts. Results from this study show that connectivity differences associated with PRS predate significant alcohol use, and sensitivity analyses did not detect a significant effect of drinking (maximum number of drinks consumed in a typical week, last 12 months) or DSM-5 AUD (lifetime diagnosis) on the PRS-EEG coherence association (Supplementary Tables S5 and S6). This suggests that the connectivity differences observed in this study are likely markers of risk, rather than a consequence of heavy alcohol use. Interestingly, the influence of PRS on EEG connectivity differed among those with and without AUD (lifetime diagnosis), suggesting that the PRS has a greater influence on alpha EEG coherence throughout the brain at ages 18–24 in those with AUD, in contrast to a more limited influence on posterior alpha EEG coherence at ages 24–31 in those without AUD (Supplementary Figure S3). However, future work is needed to better understand these observed differences in the context of the many factors that differ between the two groups, including drinking behavior, mean level of DSM-IV AD PRS, psychosocial influences, etc.

Gender differences in resting EEG coherence have been observed in previous studies [35,36]. However, prior studies examining sex differences in EEG coherence across development have had relatively small sample sizes (*N* < 100) and have largely comprised cross-sectional studies of individuals unaffected with AUD or other neuropsychiatric disorders. In the single study of typical development in 40 boys and 40 girls ages 8–12 [23], there was evidence of resting state EEG coherences systematically developing across this age range, especially in long-range electrode pairs; all effects varied by gender, brain region, and frequency band, underscoring the complexity of neural development and clear differences in these processes among males and females. Barry et al. [23] also found that females seemed to “lag behind” males in EEG coherence development. Interestingly, in this study we did not find significant phenotypic differences between males and females in EEG coherence despite the striking differences observed in the influence of AD PRS on EEG coherence. Given the phenotypic sex differences observed in previous work and the striking sex differences observed in the PRS-EEG coherence associations observed in the current study, future work is clearly needed in this area.

### Limitations and Future Directions

These findings should be considered within the context of certain limitations. First and most importantly is that this study only included individuals of European ancestry, due to the majority European ancestral composition of the discovery GWAS from which the PRS were derived from, as cross-ancestry predictions are at risk for producing highly biased estimates. A future direction of this work is to create PRS derived from the Million Veteran Program GWAS of AUD, which includes considerable numbers of individuals of African and Hispanic/Latino ancestries, despite the sex-imbalance noted above. While this is the largest study of EEG coherence in adolescence and young adults conducted to date, larger sample sizes are still needed to confirm both trajectories of EEG coherence and associations with alcohol use behavior and PRS. In addition, it will be important for future work to pair EEG studies with fMRI to combine the advantages of the superior spatial resolution of MRI and the superior temporal resolution of EEG, to better understand findings from both modes of measuring functional connectivity. Finally, future studies will directly compare PRS for AUD with PRS for alcohol consumption to further disentangle the effects of genetic risk for AUD on brain function.

## 5. Conclusions and Significance

This is the first study to demonstrate the influence of polygenic risk for alcohol dependence on EEG coherence, an important marker of cognitive functioning and AUD, alongside neuropsychological performance, and lifetime alcohol and opioid use disorders. Findings from this study demonstrate clear differences among males and females in the association of polygenic risk for alcohol dependence and neural connectivity across adolescent and young adult development. These findings also suggest that patterns of polygenic association with neural connectivity differ with respect to age among those with and without lifetime AUD. Results from this study show that connectivity differences associated with PRS predate significant alcohol use, suggesting that the EEG coherence is likely a marker of risk, rather than a consequence of heavy alcohol use. Therefore, the cognitive deficits reflected by EEG coherence levels and neuropsychological performance may be one mechanism by which polygenic risk increases risk for AUD. While polygenic risk scores for AUD have yet to reach the point of clinical utility (i.e., family history of AUD remains a stronger predictor than any PRS), larger and more diverse GWAS of AUD currently underway hold promise for the future. Well-characterized, longitudinal, and diverse samples are needed to ‘translate’ results emerging from GWAS, particularly with respect to brain function, which might serve as a pathway for genetic vulnerability for AUD to be expressed. While additional research is needed in this area, this study is a first step in understanding one potential neural mechanism linking genetic variants emerging from GWAS to neural development and may help to identify specific points in adolescence or young adulthood when neurocognitive prevention and intervention efforts may be most effective.

## Figures and Tables

**Table 1:** Descriptive characteristics in the analytic sample.

	Prospective Study EEG Subsample of European Ancestry
Genotyped (*N*)	1426
Female (%)	51.6%
Mean Age (SD)	17.7 (7.4)
Self-reported as ‘White’ (%)	98.1%
Self-reported as ‘Black’ (%)	0.1
Self-reported as ‘Latin/Hispanic’ (%)	0.1
Self-reported as ‘Other’ (%)	4.4
Family History of AUD (%)	41.1
Ever Drinkers (%)	71.8
DSM-5 AUD (%)	35.4
DSM-5 Cannabis Use Disorder, lifetime (%)	23.4
DSM-5 Cocaine Use Disorder, lifetime (%)	4.2
DSM-5 Opioid Use Disorder, lifetime (%)	5.3

**Table 2:** First quartile of association beta coefficients and *p*-values of AD PRS (*p*-threshold < 0.001) with low theta and high alpha EEG coherence in males ages 12–31 with −log10 transformation. All values in bold meet the false discovery rate criterion of 10^−4^**.**

		Ages 12–17		Ages 18–25		Ages 26–31	
		Low theta	High alpha	Low theta	High alpha	Low theta	High alpha
**Pair Frontal central sagittal**		beta (−log10 *p*-value)		beta (−log10 *p*-value)		beta (−log10 *p*-value)	
1	F8-T8--F7-T7	0.01 (1.55)	0.01 (0.58)	0.01 (0.70)	0.01 (2.25)	(0.01)1.04	0.02 (4.12)
2	F4-C4--F3-C3	0.01 (1.09)	0.02 (2.56)	0.03 (6.15 *)	**0.03 (4.98)**	0.02 (3.77)	**0.03 (5.66)**
3	F3-C3--F8-T8	0.01 (1.56)	0.00 (0.54)	0.01 (1.98)	0.01 (1.81)	0.01 (1.43)	**0.03 (5.71)**
4	F4-C4--F7-T7	0.01 (0.73)	0.01 (0.86)	0.01 (1.87)	0.02 (2.90)	0.01 (1.08)	0.02 (3.03)
5	F3-C3--F7-T7	0.01 (0.55)	0.00 (0.64)	**0.03 (4.25)**	0.05 (7.62 *)	0.02 (2.08)	0.04 (6.95 *)
6	F4-C4--F8-T8	0.01 (1.15)	0.01 (1.18)	0.01 (0.57)	0.01 (0.89)	0.02 (1.83)	0.03 (3.73)
7	FZ-CZ--F7-T7	0.0 (0.36)	0.01 (1.10)	0.01 (2.11)	**0.02 (4.65)**	0.01 (1.46)	**0.02 (4.04)**
8	FZ-CZ--F3-C3	0.02 (2.37)	**0.03 (5.06)**	0.05 (9.30 **)	0.05 (9.60 **)	0.03 (3.48)	**0.03 (4.30)**
9	FZ-CZ--F8-T8	0.01 (1.14)	0.01 (1.23)	0.00 (0.26)	0.01 (1.32)	0.01 (0.81)	0.02 (3.01)
10	FZ-CZ--F4-C4	0.01 (0.65)	0.01 (1.34)	0.03 (3.85)	0.02 (2.82)	**0.04 (5.80)**	0.04 (6.87 *)
**Central-Parietal sagittal**							
11	T8-P8--T7-P7	0.00 (0.88)	0.01 (1.34)	0.02 (7.14 *)	0.03 (6.19 *)	0.02 (6.33 *)	0.05 (10.81 ***)
12	C4-P4--C3-P3	0.01 (0.79)	0.02 (2.59)	0.03 (8.03 **)	0.04 (6.54 *)	0.03 (5.46)	**0.03 (4.23)**
13	C3-P3--T8-P8	0.00 (0.60)	0.02 (1.70)	**0.02 (5.94)**	**0.03 (4.61)**	0.01 (2.49)	0.04 (6.38*)
14	C4-P4--T7-P7	0.01 (1.19)	0.02 (1.61)	**0.02 (4.78)**	0.04 (6.48 *)	0.01 (3.54)	**0.03 (5.27)**
15	C3-P3--T7-P7	0.01 (0.73)	0.01 (1.46)	0.02 (2.10)	0.04 (8.06 **)	0.01 (0.67)	0.04 (6.55 *)
16	C4-P4--T8-P8	0.01 (1.66)	0.02 (2.25)	**0.03 (4.91)**	**0.03 (4.38)**	0.02 (3.20)	0.04 (6.14 *)
17	T7-P7--CZ-PZ	0.00 (0.57)	0.01 (1.03)	**0.02 (4.47)**	**0.04 (5.30)**	0.01 (3.42)	**0.04 (5.81)**
18	C3-P3--CZ-PZ	0.02 (2.85)	0.02 (2.56)	0.05 (10.12 ***)	0.04 (6.67 *)	0.04 (6.61 *)	**0.04 (5.58)**
19	T8-P8--CZ-PZ	0.01 (1.42)	0.01 (1.47)	0.02 (6.39 *)	**0.03 (5.58)**	0.01 (2.74)	0.03 (3.61)
20	C4-P4--CZ-PZ	0.01 (1.40)	0.02 (1.70)	**0.03 (4.24)**	**0.03 (4.09)**	**0.03 (4.76)**	0.03 (3.56)
**Parietal-Occipital sagittal**							
21	P4-O2--P3-O1	0.02 (3.72)	0.03 (3.20)	0.04 (11.65 ***)	0.05 (10.07 ***)	0.05 (10.52 ***)	0.06 (9.75 **)
**Intrahemispheric lateral**							
22	T7-C3--F7-F3	0.01 (0.99)	0.01 (0.60)	0.04 (6.19 *)	0.03 (3.76)	**0.03 (4.70)**	0.02 (2.14)
23	P7-P3--F7-F3	0.00 (0.99)	0.01 (1.14)	**0.02 (5.49)**	**0.02 (4.01)**	0.02 (2.64)	0.01 (1.61)
24	P7-P3--T7-C3	0.01 (1.76)	0.03 (3.89)	**0.03 (5.64)**	**0.04 (5.62)**	**0.03 (4.08)**	0.04 (6.11 *)
25	T8-C4--F8-F4	0.00 (0.27)	0.01 (1.15)	0.03 (3.34)	0.01 (1.09)	**0.04 (5.19)**	0.03 (3.33)
26	P8-P4--F8-F4	0.00 (0.38)	0.01 (1.68)	0.01 (2.89)	0.02 (3.57)	0.01 (2.67)	0.01 (2.37)
27	P8-P4--T8-C4	0.00 (0.72)	0.02 (1.49)	0.03 (6.39 *)	0.03 (3.17)	**0.03 (4.88)**	0.03 (2.67)

**Note**: *** *p* < 5 × 10^−10^; ** *p* < 5 × 10^−8^; * *p* < 5 × 10^−6^, *p* < 5× 10^−4^ are bolded.

**Table 3:** Association of AD PRS (*p* < 0.001) with neuropsychological performance and symptoms of DSM-5 alcohol, cannabis, cocaine, and opioid use disorder.

	DSM-IV AD PRS (*p* < 0.001)	
**Neuropsychological Performance**	Beta (Model 1)	Beta (Model 2)
TOLT Performance (number of optimal trials)	−0.097 **	−0.027 *
TOLT Speed (average trial time)	0.012	0.010
VST Backwards Visual Span	0.090 *	0.040 *
VST Forwards Visual Span	−0.123 ***	−0.071 **
**Substance Use Disorders (Lifetime)**		
DSM-5 Max Alcohol Use Disorder Symptom Count	0.087 **	0.033 *
DSM-5 Max Cannabis Use Disorder Symptom Count	0.043	0.023
DSM-5 Max Cocaine Use Disorder Symptom Count	0.037	0.017
DSM-5 Max Opioid Use Disorder Symptom Count	0.069 **	0.049 *

*** *p* < 0.0001; ** *p* < 0.001; * *p* < 0.01; Model 1 covariates: age, sex, PCs1-3, genotype array; Model 2 covariates: age, sex, PCs, genotype array, maximum number of drinks consumed in a typical week (in the past 12 months).

## Supplementary Materials

The following are available online at https://www.mdpi.com/2076-3425/9/10/280/s1. **Figure S1**. Age Distribution of the Observations in the Analytic Sample. Figure S2. Effect sizes from standardized PRS (*p* < 0.001) of high alpha (dotted lines) and low theta (solid lines) fronto-temporal (blue), tempo-parietal (green), and parietal-occipital (red) interhemispheric coherences in males from ages 12–31. Figure S3. Association (−log10 p-values) of AD PRS (three p-thresholds ranging from *p* < 0.0001, *p* < 0.001, *p* < 0.01, within each frequency band, along the x-axis) with low theta, high theta, low alpha, high alpha, low beta, mid beta and high beta EEG coherence at coherence pairs (y-axis) organized (top to bottom) from interhemispheric anterior pairs to posterior pairs, and intra-hemispheric pairs in males ages 12–18 (top panel), 18–25 (middle panel), and 25–31 (bottom panel). Figure S4. Association (-log 10 p-values) of DSM-IV AD PRS (*p* < 0.001 threshold) at coherence pairs (y-axis) organized (top to bottom) from interhemispheric anterior pairs to posterior pairs, and intra-hemispheric pairs among males with a lifetime DSM-5 alcohol use disorder (panel A) and males without lifetime DSM-5 alcohol use disorder (panel B); among those with alcohol use disorder, prominent associations with fronto-central, tempo-frontal (F3-C3--F7-T7, FZ-CZ--F3-C3), centro-parietal (C4-P4--C3-P3, C3-P3--CZ-PZ), parietal-occipital (P4-O2--P3-O1), and fronto-parietal (P8-P4--F8-F4) high alpha coherences are observed only among males from ages 18–24 (panel A), whereas prominent associations are only observed with parietal-occipital (P4-O2--P3-O1) high alpha coherences among males from ages 24–31 (panel B). Figure S5. Trajectories of mean high alpha EEG coherences in males (top panel) and females (bottom panel) from ages 12–31. Lines correspond to quartile values; 25%, 50%, 75%. Table S1. Correlations of all PRS thresholds analyzed in this study. Table S2. First Quartile of Association −log 10 *p*-values of AD PRS (*p*-threshold < 0.001) with low theta, high theta, low alpha and high alpha EEG coherence in males ages 12–31. Table S3. Association (−log10 *p*-values) of AD PRS (*p*-threshold < 0.001) with low theta and high alpha EEG coherence in females ages 12–31. Table S4. First Quartile of Male vs. Female differences −log10 *p*-values of AD PRS (*p*-threshold < 0.001) with low theta and high alpha EEG coherence in males ages 12–31. Table S5. Association (beta coefficients) of AD PRS (p-threshold < 0.001) with high alpha EEG coherence in males ages 12–31, adjusted for average maximum number of drinks consumed in a typical week (past 12 months). Table S6. Association (beta coefficients) of AD PRS (*p*-threshold < 0.001) with high alpha EEG coherence in males ages 12–31, adjusted for DSM-5 AUD (lifetime). EEG Coherence Recording & Data Reduction.
