# Supplementary material for: Association of Polygenic Liability for Alcohol Dependence and EEG Connectivity in Adolescence and Young Adulthood
Source: Brain Sci. 2019 Oct 17;9(10):280. doi: 10.3390/brainsci9100280 (PMC6826735; doi:10.3390/brainsci9100280)
Supplement: Supplementary file 1 [file brainsci-09-00280-s001.pdf]

**Supplementary**

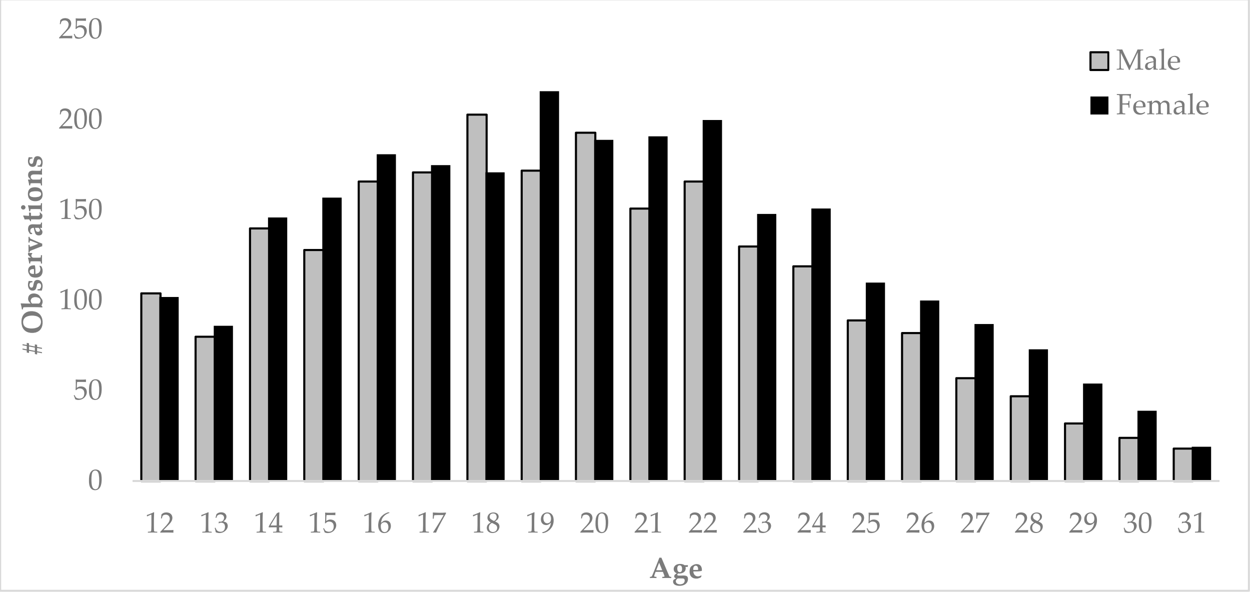


**Figure S1.** Age Distribution of the Observations in the Analytic Sample

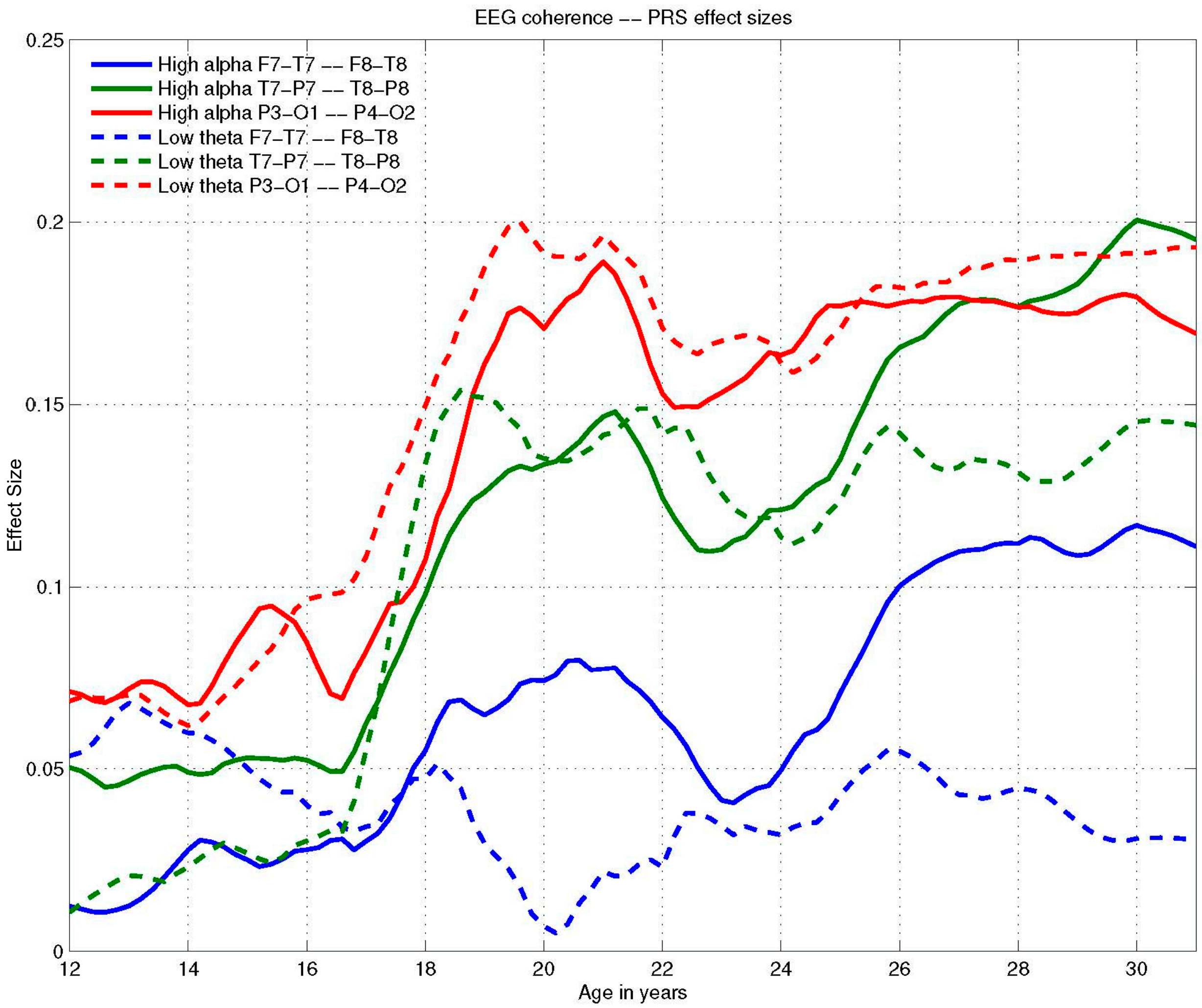


**Figure S2.** Effect sizes from standardized PRS ($p<0.001$) of high alpha (dotted lines) and low theta (solid lines) fronto-temporal (blue), tempo-parietal (green), and parietal-occipital (red) interhemispheric coherences in males from ages 12–31.

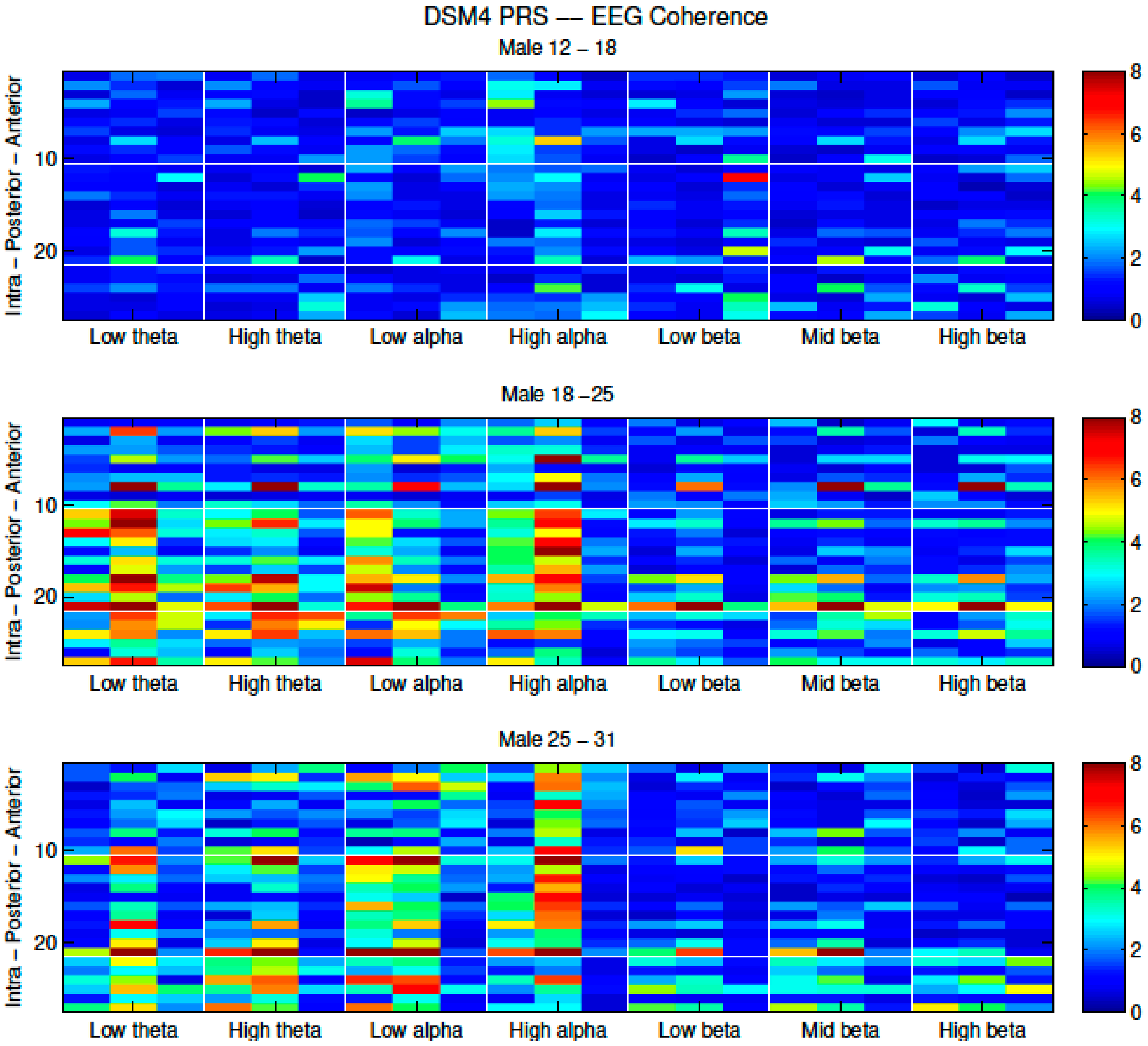


**Figure S3.** Association (−log10 p-values) of AD PRS (three p-thresholds ranging from $p<0.0001$, $p<0.001$, $p<0.01$, within each frequency band, along the x-axis) with low theta, high theta, low alpha, high alpha, low beta, mid beta and high beta EEG coherence at coherence pairs (y-axis) organized (top to bottom) from interhemispheric anterior pairs to posterior pairs, and intra-hemispheric pairs in males ages 12–18 (top panel), 18–25 (middle panel), and 25–31 (bottom panel).

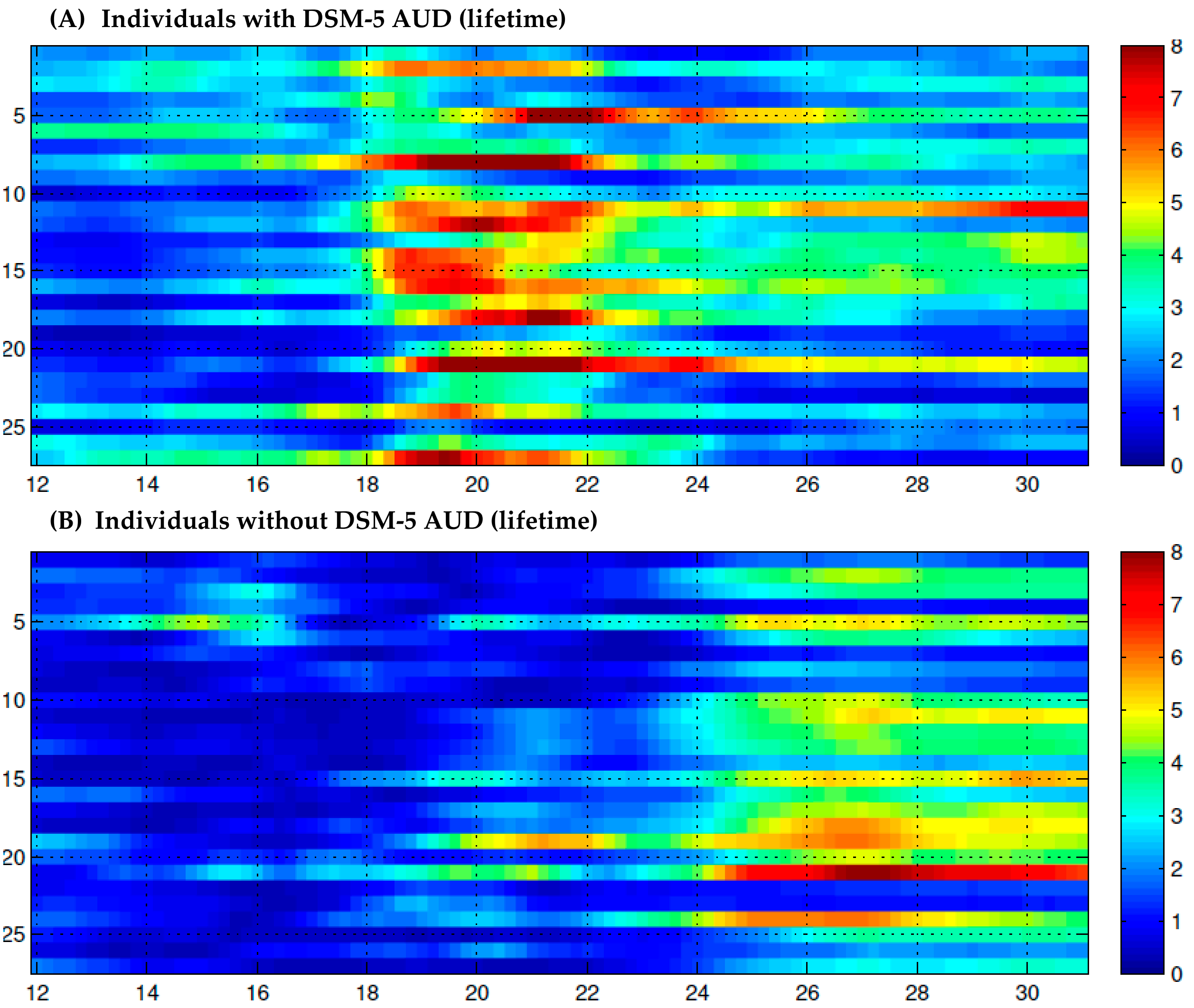


**Figure S4.** Association (-log 10 p-values) of DSM-IV AD PRS (p<0.001 threshold) at coherence pairs (y-axis) organized (top to bottom) from interhemispheric anterior pairs to posterior pairs, and intra-hemispheric pairs among males with a lifetime DSM-5 alcohol use disorder (panel A) and males without lifetime DSM-5 alcohol use disorder (panel B); among those with alcohol use disorder, prominent associations with fronto-central, tempo-frontal (F3-C3--F7-T7, FZ-CZ--F3-C3), centro-parietal (C4-P4--C3-P3, C3-P3--CZ-PZ), parietal-occipital (P4-O2--P3-O1), and fronto-parietal (P8-P4--F8-F4) high alpha coherences are observed only among males from ages 18–24 (panel A), whereas prominent associations are only observed with parietal-occipital (P4-O2--P3-O1) high alpha coherences among males from ages 24–31 (panel B).

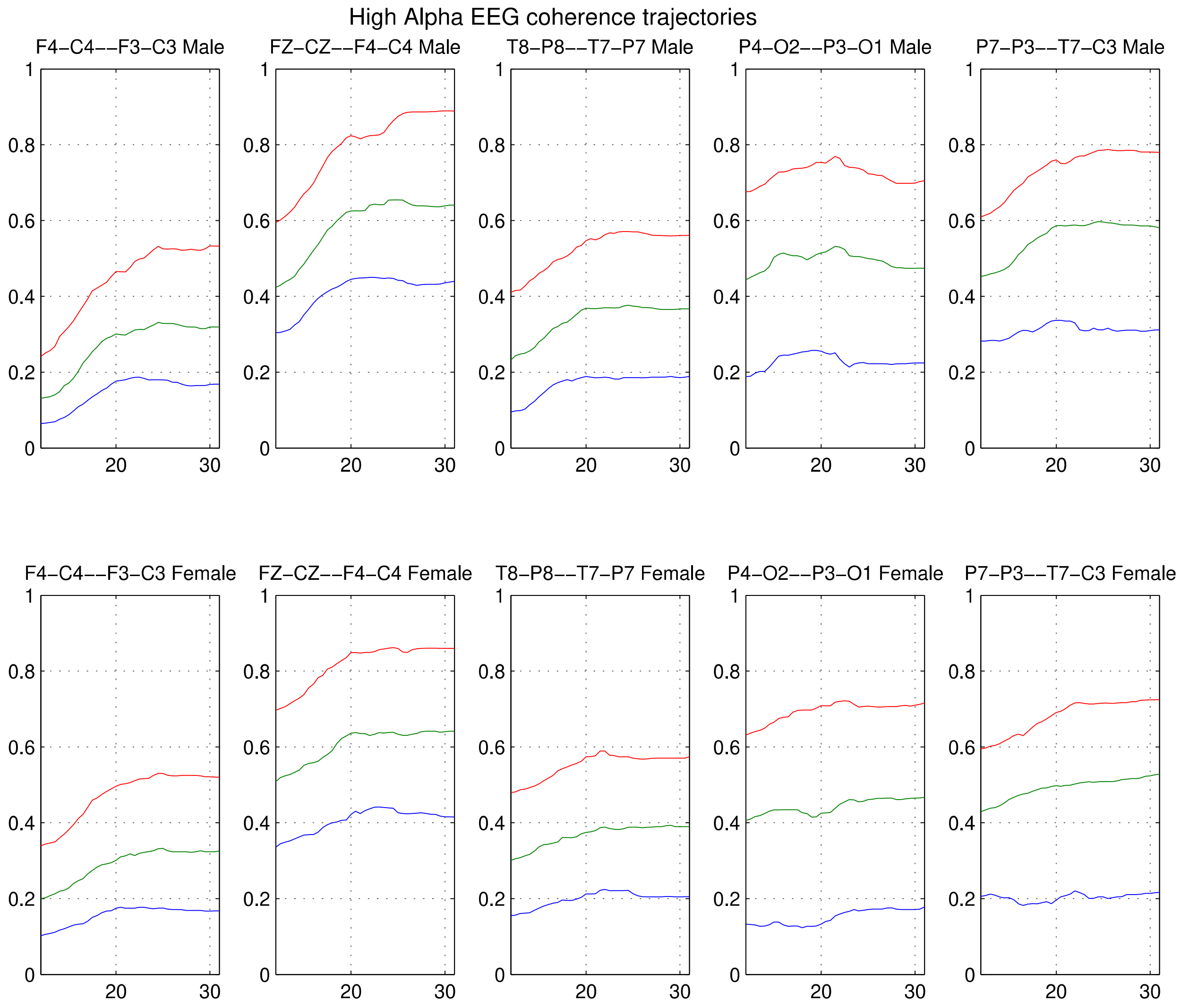


**Figure S5.** Trajectories of mean high alpha EEG coherences in males (top panel) and females (bottom panel) from ages 12–31. Lines correspond to quartile values; 25%, 50%, 75%.

**Table S1.** Correlations of all PRS thresholds analyzed in this study.

| | | ***PRS p-value thresholds*** | | | | | | | | |
|---|---|---|---|---|---|---|---|---|---|---|
| ***PRS*** | **N SNPs** | ***p*<0.0001** | ***p*<0.001** | ***p*<0.01** | ***p*<0.05** | ***p*<0.1** | *p*<0.2 | *p*<0.3 | *p*<0.4 | *p*<0.5 |
| *p*<0.0001 | 422 | 1.000 | 0.543 | 0.318 | 0.265 | 0.264 | 0.251 | 0.252 | 0.248 | 0.248 |
| *p*<0.001 | 3246 | 0.543 | 1.000 | 0.663 | 0.572 | 0.541 | 0.515 | 0.513 | 0.507 | 0.507 |
| *p*<0.01 | 25558 | 0.318 | 0.663 | 1.000 | 0.869 | 0.830 | 0.804 | 0.798 | 0.793 | 0.792 |
| *p*<0.05 | 102706 | 0.265 | 0.572 | 0.869 | 1.000 | 0.966 | 0.943 | 0.934 | 0.931 | 0.929 |
| *p*<0.1 | 183848 | 0.264 | 0.541 | 0.830 | 0.966 | 1.000 | 0.981 | 0.974 | 0.971 | 0.970 |
| *p*<0.2 | 319986 | 0.251 | 0.515 | 0.804 | 0.943 | 0.981 | 1.000 | 0.994 | 0.992 | 0.990 |
| *p*<0.3 | 434616 | 0.252 | 0.513 | 0.798 | 0.934 | 0.974 | 0.994 | 1.000 | 0.998 | 0.997 |
| *p*<0.4 | 531982 | 0.248 | 0.507 | 0.793 | 0.931 | 0.971 | 0.992 | 0.998 | 1.000 | 0.999 |
| *P*<0.5 | 613940 | 0.248 | 0.507 | 0.792 | 0.929 | 0.970 | 0.990 | 0.997 | 0.999 | 1.000 |

Note: N SNPs indicates the number of single nucleotide polymorphisms (SNPS) included in each Polygenic Risk Score (PRS) at differing p-value thresholds based on Walters et al., 2019.

**Table S2.** First Quartile of Association -log 10 p-values of AD PRS (*p*-threshold <0.001) with low theta, high theta, low alpha and high alpha EEG coherence in males ages 12–31.

| | | Ages 12-17 | | | | Ages 18-25 | | | | Ages 26-31 | | | |
|---|---|---|---|---|---|---|---|---|---|---|---|---|---|
| | | Low theta | High theta | Low alpha | High alpha | Low theta | High theta | Low alpha | High alpha | Low theta | High theta | Low alpha | High alpha |
| | Frontal central sagittal | | −log10 p-value | | | | −log10 p-value | | | | -log10 p-value | | |
| 1 | F8-T8--F7-T7 | 1.55 | 1.47 | 0.71 | 0.58 | 0.70 | 0.67 | 0.58 | 2.25 | 1.04 | 2.05 | 1.67 | 4.12 |
| 2 | F4-C4--F3-C3 | 1.09 | 0.73 | 1.40 | 2.56 | **6.15** | 4.96 | 4.09 | 4.98 | 3.77 | 4.81 | 4.68 | **5.66** |
| 3 | F3-C3--F8-T8 | 1.56 | 0.91 | 0.84 | 0.54 | 1.98 | 1.30 | 1.23 | 1.81 | 1.43 | 2.99 | **5.87** | **5.71** |
| 4 | F4-C4--F7-T7 | 0.73 | 0.42 | 0.86 | 0.86 | 1.87 | 1.02 | 1.21 | 2.90 | 1.08 | 0.46 | 0.95 | 3.03 |
| 5 | F3-C3--F7-T7 | 0.55 | 0.12 | 0.36 | 0.64 | 4.25 | 3.90 | 4.81 | **7.62** | 2.08 | 2.06 | 3.77 | **6.95** |
| 6 | F4-C4--F8-T8 | 1.15 | 0.86 | 1.32 | 1.18 | 0.57 | 0.33 | 0.24 | 0.89 | 1.83 | 1.33 | 1.26 | 3.73 |
| 7 | FZ-CZ--F7-T7 | 0.36 | 0.45 | 1.04 | 1.10 | 2.11 | 1.17 | 1.13 | 4.65 | 1.46 | 0.98 | 1.35 | 4.04 |
| 8 | FZ-CZ--F3-C3 | 2.37 | 2.12 | 3.62 | **5.06** | **9.30**** | **7.81** | **6.77** | **9.60**** | 3.48 | 3.71 | 3.49 | 4.30 |
| 9 | FZ-CZ--F8-T8 | 1.14 | 0.87 | 1.51 | 1.23 | 0.26 | 0.43 | 0.24 | 1.32 | 0.81 | 0.50 | 0.93 | 3.01 |
| 10 | FZ-CZ--F4-C4 | 0.65 | 0.57 | 1.22 | 1.34 | 3.85 | 2.33 | 1.71 | 2.82 | **5.80** | 4.92 | 4.21 | **6.87** |
| | Central-Parietal sagittal | | | | | | | | | | | | |
| 11 | T8-P8--T7-P7 | 0.88 | 0.73 | 0.77 | 1.34 | **7.14** | 3.81 | 3.02 | **6.19** | **6.33** | **7.97** | **8.21**** | **10.81**** |
| 12 | C4-P4--C3-P3 | 0.79 | 0.87 | 0.25 | 2.59 | **8.03**** | **6.27** | 3.64 | **6.54** | **5.46** | 3.89 | 4.33 | 4.23 |
| 13 | C3-P3--T8-P8 | 0.60 | 0.27 | 0.37 | 1.70 | **5.94** | 2.98 | 0.87 | 4.61 | 2.49 | 2.12 | 3.40 | **6.38** |
| 14 | C4-P4--T7-P7 | 1.19 | 0.98 | 0.42 | 1.61 | 4.78 | 2.57 | 2.35 | **6.48** | 3.54 | 2.99 | 3.81 | **5.27** |
| 15 | C3-P3--T7-P7 | 0.73 | 0.61 | 0.97 | 1.46 | 2.10 | 1.82 | 2.03 | **8.06**** | 0.67 | 0.87 | 2.24 | **6.55** |
| 16 | C4-P4--T8-P8 | 1.66 | 0.77 | 0.53 | 2.25 | 4.91 | 3.90 | 3.17 | 4.38 | 3.20 | 2.09 | 3.70 | **6.14** |
| 17 | T7-P7--CZ-PZ | 0.57 | 0.81 | 1.31 | 1.03 | 4.47 | 2.32 | 1.82 | **5.30** | 3.42 | 2.28 | 3.46 | **5.81** |
| 18 | C3-P3--CZ-PZ | 2.85 | 1.58 | 0.78 | 2.56 | **10.12**** | **7.38** | 4.77 | **6.67** | **6.61** | **5.37** | **5.05** | **5.58** |
| 19 | T8-P8--CZ-PZ | 1.42 | 0.58 | 0.21 | 1.47 | **6.39** | **5.30** | 1.64 | **5.58** | 2.74 | 1.57 | 1.68 | 3.61 |
| 20 | C4-P4--CZ-PZ | 1.40 | 0.99 | 0.62 | 1.70 | 4.24 | 3.77 | 3.39 | 4.09 | 4.76 | 4.79 | 4.40 | 3.56 |
| | Parietal-Occipital sagittal | | | | | | | | | | | | |
| 21 | P4-O2--P3-O1 | 3.72 | 3.10 | 2.74 | 3.20 | **11.65**** | **11.06**** | **10.28**** | **10.07**** | **10.52**** | **11.77**** | **12.56**** | **9.75**** |
| | Intrahemispheric lateral | | | | | | | | | | | | |
| 22 | T7-C3--F7-F3 | 0.99 | 0.58 | 0.44 | 0.60 | **6.19** | **6.21** | **6.18** | 3.76 | 4.70 | 3.99 | 3.57 | 2.14 |
| 23 | P7-P3--F7-F3 | 0.99 | 0.44 | 0.32 | 1.14 | **5.49** | **5.69** | 4.68 | 4.01 | 2.64 | 4.24 | 2.79 | 1.61 |
| 24 | P7-P3--T7-C3 | 1.76 | 1.69 | 1.11 | 3.89 | **5.64** | **6.13** | **5.20** | **5.62** | 4.08 | **5.94** | **6.07** | **6.11** |
| 25 | T8-C4--F8-F4 | 0.27 | 0.49 | 0.35 | 1.15 | 3.34 | 1.93 | 2.06 | 1.09 | **5.19** | **5.59** | **6.68** | **3.33** |
| 26 | P8-P4--F8-F4 | 0.38 | 0.36 | 0.83 | 1.68 | 2.89 | 2.32 | 2.95 | 3.57 | 2.67 | 2.88 | 2.73 | 2.37 |
| 27 | P8-P4--T8-C4 | 0.72 | 0.51 | 0.25 | 1.49 | **6.39** | 3.83 | 3.69 | 3.17 | 4.88 | 3.88 | 3.71 | 2.67 |

Note: Coherence pairs with p-values<5x10$^{-8}$ are indicated with **; p-values<5x10$^{-5}$ are bolded;

**Table S3.** Association (−log10 p-values) of AD PRS (*p*-threshold <0.001) with low theta and high alpha EEG coherence in females ages 12–31.

| | | **Ages 12–17** | | **Ages 18–25** | | **Ages 26–31** | |
|---|---|---|---|---|---|---|---|
| | | **Low theta** | **High alpha** | **Low theta** | **High alpha** | **Low theta** | **High alpha** |
| | Frontal central sagittal | −log10 *p*-value | | −log10 *p*-value | | −log10 *p*-value | |
| 1 | F8-T8--F7-T7 | 1.40 | 0.57 | 3.03 | 1.33 | 1.18 | 0.90 |
| 2 | F4-C4--F3-C3 | 1.25 | 1.11 | 0.93 | 1.77 | 0.57 | 1.20 |
| 3 | F3-C3--F8-T8 | 0.69 | 0.66 | 3.38 | 0.91 | 1.91 | 1.12 |
| 4 | F4-C4--F7-T7 | **4.28** | 1.71 | 1.08 | 1.66 | 1.18 | 2.48 |
| 5 | F3-C3--F7-T7 | 2.98 | 1.67 | 2.92 | 1.14 | 3.62 | 0.99 |
| 6 | F4-C4--F8-T8 | 1.46 | 0.82 | 0.69 | 1.32 | 0.47 | 0.44 |
| 7 | FZ-CZ--F7-T7 | 1.69 | 0.97 | 1.32 | 2.25 | 0.57 | 0.94 |
| 8 | FZ-CZ--F3-C3 | 3.02 | 0.51 | 1.61 | 1.62 | 1.41 | 2.32 |
| 9 | FZ-CZ--F8-T8 | 0.60 | 1.62 | 2.14 | 1.26 | 0.94 | 1.15 |
| 10 | FZ-CZ--F4-C4 | 1.65 | 0.91 | 2.15 | 1.76 | 1.77 | 2.63 |
| | Central-Parietal sagittal | | | | | | |
| 11 | T8-P8--T7-P7 | 0.51 | 1.57 | 0.73 | 1.68 | 1.49 | 3.38 |
| 12 | C4-P4--C3-P3 | 0.65 | 0.65 | 0.83 | 1.13 | 0.51 | 1.28 |
| 13 | C3-P3--T8-P8 | 1.87 | 0.85 | 1.56 | 0.51 | 1.63 | 1.24 |
| 14 | C4-P4--T7-P7 | 0.81 | 1.65 | 1.00 | 1.59 | 2.90 | 3.01 |
| 15 | C3-P3--T7-P7 | 1.14 | 0.90 | 3.83 | 1.27 | **5.98** | 0.73 |
| 16 | C4-P4--T8-P8 | 0.95 | 1.83 | 1.33 | 3.36 | 3.22 | 3.61 |
| 17 | T7-P7--CZ-PZ | 0.64 | 1.46 | 0.69 | 0.97 | 0.64 | 1.49 |
| 18 | C3-P3--C.Z-PZ | 1.71 | 1.34 | 0.62 | 1.75 | 1.48 | 1.24 |
| 19 | T8-P8--CZ-PZ | 0.95 | 1.12 | 1.29 | 0.79 | 1.47 | 2.61 |
| 20 | C4-P4--CZ-PZ | 0.79 | 0.92 | 2.43 | 1.18 | 1.66 | 1.92 |
| | Parietal-Occipital sagittal | | | | | | |
| 21 | P4-O2--P3-O1 | 0.64 | 3.07 | 3.07 | 1.77 | 0.80 | 0.71 |
| | Intrahemispheric lateral | | | | | | |
| 22 | T7-C3--F7-F3 | 1.19 | 2.34 | 0.49 | 0.83 | 1.24 | 0.72 |
| 23 | P7-P3--F7-F3 | 1.89 | 1.16 | 1.14 | 1.19 | 1.28 | 2.97 |
| 24 | P7-P3--T7-C3 | 0.69 | 1.83 | 0.53 | 1.53 | 1.44 | 2.95 |
| 25 | T8-C4--F8-F4 | 2.21 | 0.96 | 0.51 | 1.21 | 1.81 | 1.78 |
| 26 | P8-P4--F8-F4 | 1.60 | 1.21 | 1.91 | 1.04 | 0.78 | 2.08 |
| 27 | P8-P4--T8-C4 | 1.56 | 2.30 | 1.26 | 2.72 | 0.79 | 2.88 |

Note: $p<5x10^{-4}$ are bolded.

**Table S4.** First Quartile of Male vs. Female differences −log10 *p*-values of AD PRS (*p*-threshold <0.001) with low theta and high alpha EEG coherence in males ages 12–31.

| | | **Ages 12–17** | | **Ages 18–25** | | **Ages 26–31** | |
|---|---|---|---|---|---|---|---|
| | | **Low theta** | **High alpha** | **Low theta** | **High alpha** | **Low theta** | **High alpha** |
| Frontal central sagittal | | -log10 *p*-value | | -log10 *p*-value | | -log10 *p*-value | |
| 1 | F8-T8--F7-T7 | 1.46 | 1.64 | 0.77 | 1.89 | 0.44 | 2.89 |
| 2 | F4-C4--F3-C3 | 0.34 | 2.26 | 2.1 | 3.75 | 1.47 | 2.13 |
| 3 | F3-C3--F8-T8 | 2.04 | 1.92 | 0.36 | 1.03 | 0.32 | 1.22 |
| 4 | F4-C4--F7-T7 | 2.67 | 2.75 | 1.19 | 2.34 | 0.78 | 1.23 |
| 5 | F3-C3--F7-T7 | 0.80 | 0.81 | 2.23 | 3.06 | 1.58 | 2.46 |
| 6 | F4-C4--F8-T8 | 0.77 | **4.95** | 0.45 | 0.91 | 0.57 | 1.03 |
| 7 | FZ-CZ--F7-T7 | 1.23 | 2.08 | 1.26 | 1.37 | 1.3 | 2.72 |
| 8 | FZ-CZ--F3-C3 | 0.26 | 2.76 | **4.76** | **6.03**** | 2.03 | 2.18 |
| 9 | FZ-CZ--F8-T8 | 1.44 | 2.07 | 1.53 | 0.73 | 1.32 | 1.47 |
| 10 | FZ-CZ--F4-C4 | 0.56 | 0.8 | 2.49 | 2.73 | 3.49 | **5.67** |
| Central-Parietal sagittal | | | | | | | |
| 11 | T8-P8--T7-P7 | 0.59 | 1.40 | 3.03 | **4.99** | 1.28 | 3.68 |
| 12 | C4-P4--C3-P3 | 0.75 | 3.27 | 2.63 | 2.76 | 1.70 | 2.23 |
| 13 | C3-P3--T8-P8 | 1.27 | 2.37 | 1.31 | 3.98 | 1.37 | 1.96 |
| 14 | C4-P4--T7-P7 | 2.35 | 2.46 | 1.69 | **4.97** | 0.58 | 1.92 |
| 15 | C3-P3--T7-P7 | 0.90 | 2.25 | 1.44 | **5.43** | 0.84 | 3.27 |
| 16 | C4-P4--T8-P8 | 2.18 | **4.07** | 2.66 | 2.83 | 1.58 | **4.64** |
| 17 | T7-P7--CZ-PZ | 1.63 | 1.9 | 3.95 | **5.05** | 2.04 | **5.15** |
| 18 | C3-P3--CZ-PZ | 0.59 | 2.38 | **5.24** | 4.13 | **4.43** | **4.86** |
| 19 | T8-P8--CZ-PZ | **4.34** | 2.12 | 3.31 | **4.75** | 0.50 | 1.94 |
| 20 | C4-P4--CZ-PZ | 2.43 | 3.05 | 2.92 | 2.01 | 3.22 | 2.73 |
| Parietal-Occipital sagittal | | | | | | | |
| 21 | P4-O2--P3-O1 | 2.62 | 3.08 | **7.05**** | **9.22***** | **7.79**** | **6.54**** |
| Intrahemispheric lateral | | | | | | | |
| 22 | T7-C3--F7-F3 | 0.22 | 1.49 | **4.90** | **4.98** | **4.43** | 3.02 |
| 23 | P7-P3--F7-F3 | 1.92 | 0.85 | 2.46 | 2.89 | 1.78 | 0.87 |
| 24 | P7-P3--T7-C3 | 0.75 | **4.40** | 3.96 | **7.01**** | **4.04** | **5.95** |
| 25 | T8-C4--F8-F4 | 0.18 | 2.80 | 2.13 | 3.50 | 3.60 | **4.75** |
| 26 | P8-P4--F8-F4 | 0.18 | **4.09** | 1.23 | **6.42**** | 3.50 | 1.67 |
| 27 | P8-P4--T8-C4 | 0.56 | 2.21 | **4.79** | **4.45** | **6.57**** | 3.48 |

Note: ***$p$<5x10-8; **$p$<5x10-6; $p$<5x10-4 are bolded.

**Table S5**. Association (beta coefficients) of AD PRS (*p*-threshold <0.001) with high alpha EEG coherence in males ages 12–31, adjusted for average maximum number of drinks consumed in a typical week (past 12 months)

| | | **Ages 12–17** | **Ages 18–25** | **Ages 26–31** |
|---|---|---|---|---|
| | | High alpha | High alpha | High alpha |
| | Frontal central sagittal | B | B | B |
| 1 | F8-T8--F7-T7 | 0.006 | 0.012 | 0.023 |
| 2 | F4-C4--F3-C3 | 0.014 | **0.030** | **0.036** |
| 3 | F3-C3--F8-T8 | 0.003 | 0.013 | **0.030** |
| 4 | F4-C4--F7-T7 | 0.007 | 0.015 | 0.020 |
| 5 | F3-C3--F7-T7 | -0.002 | **0.044*** | **0.045**** |
| 6 | F4-C4--F8-T8 | 0.007 | 0.011 | **0.035** |
| 7 | FZ-CZ--F7-T7 | 0.010 | 0.022 | 0.024 |
| 8 | FZ-CZ--F3-C3 | **0.032** | **0.048**** | **0.034** |
| 9 | FZ-CZ--F8-T8 | 0.006 | 0.011 | 0.023 |
| 10 | FZ-CZ--F4-C4 | 0.012 | 0.026 | **0.049**** |
| | Central-Parietal sagittal | | | |
| 11 | T8-P8--T7-P7 | 0.012 | **0.033** | **0.051***** |
| 12 | C4-P4--C3-P3 | 0.017 | **0.040*** | **0.033** |
| 13 | C3-P3--T8-P8 | 0.010 | 0.029 | **0.040*** |
| 14 | C4-P4--T7-P7 | 0.016 | **0.034** | **0.036** |
| 15 | C3-P3--T7-P7 | 0.017 | **0.042*** | **0.039** |
| 16 | C4-P4--T8-P8 | 0.017 | **0.032** | **0.043*** |
| 17 | T7-P7--CZ-PZ | 0.013 | **0.032** | **0.041*** |
| 18 | C3-P3--C.Z-PZ | 0.020 | **0.042*** | **0.041*** |
| 19 | T8-P8--CZ-PZ | 0.009 | **0.032** | **0.032** |
| 20 | C4-P4--CZ-PZ | 0.018 | **0.030** | **0.034** |
| | Parietal-Occipital sagittal | | | |
| 21 | P4-O2--P3-O1 | 0.024 | **0.053***** | **0.054***** |
| | Intrahemispheric lateral | | | |
| 22 | T7-C3--F7-F3 | 0.007 | 0.027 | 0.022 |
| 23 | P7-P3--F7-F3 | 0.008 | 0.018 | 0.014 |
| 24 | P7-P3--T7-C3 | **0.030** | **0.035** | **0.039** |
| 25 | T8-C4--F8-F4 | 0.008 | 0.014 | **0.031** |
| 26 | P8-P4--F8-F4 | 0.008 | 0.018 | 0.016 |
| 27 | P8-P4--T8-C4 | 0.015 | 0.025 | 0.025 |

Note: ***$p$<5x10$^{-10}$; ** $p$<5x10$^{-8}$; *$p$<5x10$^{-6}$; $p$<5x10$^{-4}$ are bolded.

**Table S6**. Association (beta coefficients) of AD PRS (*p*-threshold <0.001) with high alpha EEG coherence in males ages 12–31, adjusted for DSM-5 AUD (lifetime)

| | | **Ages 12–17** | **Ages 18–25** | **Ages 26–31** |
|---|---|---|---|---|
| | | High alpha | High alpha | High alpha |
| | Frontal central sagittal | B | B | B |
| 1 | F8-T8--F7-T7 | 0.006 | 0.012 | 0.022 |
| 2 | F4-C4--F3-C3 | 0.014 | **0.030** | **0.036** |
| 3 | F3-C3--F8-T8 | 0.003 | 0.013 | **0.030** |
| 4 | F4-C4--F7-T7 | 0.006 | 0.015 | 0.019 |
| 5 | F3-C3--F7-T7 | -0.003 | **0.045**** | **0.044*** |
| 6 | F4-C4--F8-T8 | 0.006 | 0.012 | **0.036** |
| 7 | FZ-CZ--F7-T7 | 0.010 | 0.023 | 0.025 |
| 8 | FZ-CZ--F3-C3 | **0.032** | **0.048**** | **0.034** |
| 9 | FZ-CZ--F8-T8 | 0.006 | 0.011 | 0.023 |
| 10 | FZ-CZ--F4-C4 | 0.011 | 0.027 | **0.050***** |
| | Central-Parietal sagittal | | | |
| 11 | T8-P8--T7-P7 | 0.011 | **0.033** | **0.051***** |
| 12 | C4-P4--C3-P3 | 0.017 | **0.040*** | **0.035** |
| 13 | C3-P3--T8-P8 | 0.010 | 0.029 | **0.041*** |
| 14 | C4-P4--T7-P7 | 0.016 | **0.034** | **0.037** |
| 15 | C3-P3--T7-P7 | 0.017 | **0.042*** | **0.040*** |
| 16 | C4-P4--T8-P8 | 0.017 | **0.033** | **0.043*** |
| 17 | T7-P7--CZ-PZ | 0.013 | **0.033** | **0.042*** |
| 18 | C3-P3--C.Z-PZ | 0.020 | **0.043*** | **0.044*** |
| 19 | T8-P8--CZ-PZ | 0.009 | **0.032** | **0.032** |
| 20 | C4-P4--CZ-PZ | 0.018 | **0.031** | **0.035** |
| | Parietal-Occipital sagittal | | | |
| 21 | P4-O2--P3-O1 | 0.023 | **0.054***** | **0.055***** |
| | Intrahemispheric lateral | | | |
| 22 | T7-C3--F7-F3 | 0.007 | 0.027 | 0.022 |
| 23 | P7-P3--F7-F3 | 0.007 | 0.019 | 0.013 |
| 24 | P7-P3--T7-C3 | **0.030** | **0.035** | **0.040*** |
| 25 | T8-C4--F8-F4 | 0.008 | 0.014 | **0.033** |
| 26 | P8-P4--F8-F4 | 0.008 | 0.017 | 0.017 |
| 27 | P8-P4--T8-C4 | 0.015 | 0.025 | 0.027 |

Note: ***$p<5\times10^{-10}$; ** $p<5\times10^{-8}$; *$p<5\times10^{-6}$; $p<5\times10^{-4}$ are bolded.

**Supplemental Materials**

*EEG Coherence Recording & Data Reduction*

All seven sites used the same experimental procedures and EEG acquisition hardware and software. Each subject wore a fitted electrode cap (Electro-Cap International Inc.; Eaton, OH) using the 19-channel montage as specified according to the 10–20 International system [FP1, FP2, F7, F3, FZ, F4, F8, T7, C3, CZ, C4, T8, P7, P3, PZ, P4, P8, O1, O2]. The nose served as reference and the ground electrode was placed on the forehead. Electrode impedances were always maintained below 5 kOhm. The electrooculogram (EOG) was recorded from electrodes placed supraorbitally at the outer canthus of the eye. Vertical and horizontal eye movements were monitored to perform ocular artifact correction. EEG was recorded with the subjects seated comfortably in a dimly lit sound-attenuated temperature-regulated booth (Industrial Acoustics Company; Bronx, NY). They were instructed to keep their eyes closed and remain relaxed. Subjects were also cautioned not to fall asleep. Electrical activity was amplified 10,000 times by Sensorium EPA-2 Electrophysiology amplifiers (Charlotte, VT), or Neuroscan amplifiers, (Compumedics Limited; El Paso, TX) with a bandpass between 0.02 Hz to 50 Hz and recorded using the COGA software system (Neurodynamics Laboratory, SUNY Downstate Medical Center) running on Concurrent 5550 computers (Concurrent Computer Corporation, Atlanta, GA) or the Neuroscan software system (Compumedics Limited; El Paso, TX) running on i86 PCs. The sampling rate was 256 Hz and the activity was recorded for 4.25 minutes (256 seconds).

EEG analysis was performed at SUNY Downstate Medical Center. A continuous interval comprising 256 seconds of EEG data was used for analysis. Offline raw data were subjected to wavelet filtering and reconstruction to reduce high and low frequencies (Bruce and Gao, 1994; Strang and Nguyen, 1996). The s12 wavelet was used to perform a six-level analysis, and the output signal was reconstructed using levels d6 through d3. This procedure is roughly equivalent to applying a band pass filter with a range of 2–64 Hz to the data. Subsequently, eye movements were removed by use of a frequency domain method developed by Gasser (Gasser et al., 1985, 1986). This method subtracts a portion of observed ocular activity from observed EEG to obtain the true EEG, based on the difference between the cross-spectral values of trials with high ocular activity and those with low ocular activity. Visual inspection of corrected data confirmed satisfactory artifact removal characteristics.

In order to improve the localization of our signals, consideration was given to both the Laplacian and bipolar data transformations. In our case, given that much of the data was recorded with the International 10–20 system of electrode placement, which offers only 19 scalp electrodes, the use of the Laplacian was ruled out. To reduce volume conduction and reference specific effects, a bipolar transformation was used. We accepted the fact that bipolar transformations might reduce coherences when the coherent activity extended across areas which were spanned by the electrodes included in the bipolar pair (Essl and Rappelsberger, 1998). The data were software transformed into 38 bipolar derivations formed by the subtraction of adjacent electrodes in both lateral and sagittal orientations (Figure 1), and analyzed in 254 overlapping 2-second epochs by use of a Fourier transform and windowed using a Hamming function to improve the accuracy of the spectral results (Hamming, 1983). (We will use the following terminology: a derivation is a single signal obtained either as the "raw" data from a single electrode or by subtracting the signals at adjacent electrodes to obtain a bipolar derivation. A coherence pair is a pair of derivations whose coherence is estimated, and called bipolar or monopolar depending on the kind of derivation used in their estimate. A bipolar coherence pair is called sagittal or lateral depending on the orientation of the two derivations, which is the same in any pair.) The disadvantage of bipolar derivations for coherence estimates is that bipolar coherence pairs are more sparsely distributed on the scalp than monopolar pairs although this is partially compensated for by the use of both sagittally and laterally oriented pairs for interhemispheric coherence. We chose both interhemispheric and intrahemispheric pairs for examination, and for the interhemispheric pairs, pairs in both sagittal and lateral orientation, forming three cases for consideration. Only symmetrically placed derivations were used for the interhemispheric coherence pairs.

$$\gamma_{ij}(f) = \frac{|G_{ij}(f)|^2}{G_{ii}(f)G_{jj}(f)}$$ lculation,

where,

$$G_{ij}(f) = \frac{1}{N}\sum_{n} X_{in}(f)X_{jn}(f)^*$$

and $X_{in}$ is the Fourier transform of the signal in channel *i* at epoch *n*, and '*' indicates the complex conjugate, was applied to each .5 Hz frequency bin, and the results were aggregated by band and divided by bandwidth. Because of the overlapping time intervals, the number of epochs used for calculating confidence intervals for the coherence estimates was set at 128. This is conservative, since the averaging across frequency bands adds a slight additional element of independence. The bands were set as follows: low theta = 3–4.5 Hz, high theta 5–7.5 Hz.
